# Safety preparedness in arab public schools

**DOI:** 10.1016/j.heliyon.2023.e19635

**Published:** 2023-09-01

**Authors:** Naof F. Al-Ansary, Mahmoud F. El-Sharkawy, Sana A. Alsulaiman

**Affiliations:** a Department of Public Health, College of Public Health, Imam Abdulrahman Bin Faisal University, Saudi Arabia; b Department of Environmental Health, College of Public Health, Imam Abdulrahman Bin Faisal University, Saudi Arabia; c Auxiliary Agency for Preventive Health, Ministry of Health, Saudi Arabia

**Keywords:** School environment, Safety preparedness, Safety criteria, Public schools, Kingdom of Saudi Arabia

## Abstract

**Background:**

A proper and adequate school environment is important for an effective learning process and for the maintenance of students' health, given that they spend a considerable amount of time at school. Safety preparedness in schools includes, for example, protection from biological, physical, and chemical risks and physical hazards associated with poor construction and maintenance practices.

**Objectives:**

This study aims to evaluate safety preparedness in girls’ public schools in the Kingdom of Saudi Arabia (KSA).

**Methods:**

Seventeen girls' schools were randomly selected in the Eastern Province of KSA. A designed checklist was used for this study, composed of two main parts. The first part included general information about the school, while the second part was composed of 6 items with a total of 58 questions, evaluating the school's safety preparedness.

**Results:**

The safety preparedness in the studied schools ranged between 70 and 90%. Some safety practices were found to be adequately applied, others were poorly applied, and certain items were completely absent. Generally, some examined schools were not compliant several safety and emergency preparedness recommendations.

**Conclusion:**

Collaboration between the School Safety Committee and schools is essential to reach a satisfactory standard in terms of school safety. Therefore, it is recommended that the School Safety Committee engages with schools more actively, especially in terms of the preparation of a school safety management plan.

## 1 Introduction

On average, students spend about 6–7 h per day in school, or 32.5 h per week, which is considered a long time. A proper and safe school environment is important to enhance the learning process and to maintain proper health conditions [1,2]. A safe school can be defined as “a school in which students can move without risk and accidents are minimal” [3]. In addition, students and school personnel should have some level of knowledge and experience in terms of responsiveness to emergency medical situations, to avoid injuries resulting from accidents that may occur in a school environment due to the absence of recommended control measures of safety preparedness [4].

Providing a safe environment in schools is a universal challenge. School administrators and emergency planners must work together to create a healthy and safe school environment through the implementation of a variety of practices, such as improving the level of awareness of school personnel and students in terms of adequate emergency planning and preparedness [5]. These practices should be observed at all levels of education (primary, intermediate, and secondary), keeping in mind that the school administration has a great responsibility in the initiation and activation of all required safety rules and protocols [6]. Safety preparedness in schools includes the provision of safe and sufficient water supply and sanitation. Moreover, it also entails protection from biological, physical, and chemical hazards, including infectious agents carried by water or air. Physical hazards in buildings are commonly associated with the absence of emergency evacuation routes, as well as poor construction and maintenance practices, such as poorly designed exits, damaged staircases, ineffective fire suppression systems, and bad lighting [5,7].

Due to cultural practices in Saudi Arabia, all male and female schools are completely segregated, including the teachers, students, and administrative staff. Although several studies have been conducted to evaluate the safety preparedness of male schools in KSA [8,9], very few and limited similar studies have been conducted for female schools. This study was aiming to evaluate the safety preparations of girls' schools in Saudi Arabia, with the intention of increasing the awareness of students and school staff towards the safety preparedness in schools.

## 2 Materials and methods

### 2.1 Study site and duration

From a total of nearly ninety girls’ public schools in the Eastern Province of Saudi Arabia, seventeen were selected (19%) using a statistical random method. The study was conducted during a period of three consecutive months (January–March 2020). Stratified sampling was used by splitting the schools into two strata (government-constructed buildings and rentals). The government-constructed buildings were designed and built by the concerned government authorities, while rental buildings are rented out by the government from private parties and used as school facilities. Based on the percentage of each type of school, fourteen government-constructed buildings and three rentals were included in this study. Details of the selected schools, including the type of school building, number of classrooms and floors, and the total number of students and staff are presented in Table 1.

**Table 1:** Characteristics of selected schools.

School No.	Type of school building	No. of classrooms	Total No. of students	Total no. of Staff	Number of floors
1	Governmental	27	492	48	2
2	Governmental	29	379	35	2
3	Governmental	55	509	57	3
4	Governmental	50	763	71	1
5	Governmental	16	149	27	2
6	Governmental	55	1035	87	3
7	Governmental	32	383	40	3
8	Governmental	24	273	39	2
9	Governmental	24	367	58	2
10	Governmental	22	283	37	3
11	Governmental	22	295	55	2
12	Governmental	32	150	33	2
13	Governmental	41	481	51	2
14	Governmental	43	265	58	3
15	Rental	17	312	37	3
16	Rental	40	518	46	4
17	Rental	24	414	37	3

### 2.2 Evaluation of the safety preparedness

There has been a lack of standard checklists to assess the safety preparedness in schools and educational facilities. For this purpose, a safety checklist was designed based on three main resources; (1) previous similar studies [10]; (2) rules and regulations of international agencies such as the New Jersey Department of Education, USA [11]; the Minnesota School Safety Center Program, USA [6]; the Vermont Department of Education, USA [12]; the Ministry of Home Affairs, India [13]; the School Safety Audit Checklist developed in the state of Delaware, USA [14]; and (3) safety guidelines implemented in Saudi Arabia [15].

The checklist designed for this study is composed of two main parts. The first part includes general information about the school being studied, such as name and level of education, location, date of establishment, number of classrooms, and number of students and employees. The second part is composed of 6 items with a total of 58 questions concerning the evaluation of the school's safety preparedness. The first item includes 13 questions evaluating the safety preparedness of the school's main building. The second item includes 7 questions evaluating the safety preparedness of classrooms. The third item includes 13 questions evaluating the safety preparedness of laboratories. The next two items (7 and 11 questions, respectively) evaluate the safety preparedness of the school canteen and the engagement of the school safety committee. The final item includes 7 questions evaluating the school emergency evacuation plan. For each question, the possible answers are: Yes, No, or Not present. The answer “No” indicates the diminished presence of the safety item, while the answer “Not present” indicates the complete absence of the item. Statistical analysis was conducted for each safety item through the calculation of mean percentages for each possible response using Microsoft Excel. Also, an overall mean for each of the six items on the checklist was calculated. Based on recorded differences between the studied schools, the following scoring system was used to evaluate the safety preparedness: Outstanding 100%–90%, Excellent 89%–80%, Very good 79%–70%, Good 69%–60%, Average 59%–50%, Poor 49%–40%, and Below the minimum standard expected 39%–0%.

The designed safety checklist was examined by an expert statistician for validity and reliability. Reliability was evaluated by comparing different versions of the same measurement, while validity was evaluated by comparing the results to other relevant data or theories. To examine the applicability of the checklist, a pilot study was conducted on two schools (other than the 17 schools comprising the study sample). The purpose of the pilot study was to test the designed checklist before the formal data collection. All study data were collected by the researchers to ensure accuracy and quality.

### Ethical approval

This study was approved by the Ethics Committee of Imam Abdulrahman bin Faisal University, with ethics approval reference No. IRB-PGS-2019-03-357. In addition, all the required approvals from the Ministry of Education (MoE) were obtained and provided to the concerned schools. Furthermore, the safety preparedness checklist was accompanied by a cover letter clarifying the objectives of the study and outlining the confidentiality statement, and the purposes for which data is collected.

### 2.3 Statistical analysis

The Microsoft Excel Software was used for descriptive statistical analyses. The frequency and mean percentages were calculated for the three possible responses for each question (Yes, No, and Not present). In addition, the overall mean percentage of each variable was calculated to evaluate the school's safety preparedness.

## 3 Results

Table 2 presents the data analysis of the first item of the checklist (safety of the school's main building), which contains 13 questions (Q1-Q13). Most of the studied schools (94%) contained schoolyards free from flammable materials (such as furniture). Nearly half of the studied schools (53%) had a total space suitable for the number of students, while most of the schools (82%) had suitable shading to protect students from sunlight, rain, and dust. Furthermore, 88% of the schools had walls free from cracks and fractures and high enough to ensure that students cannot climb or attempt to jump off the fence. In addition, 59% of the schools had emergency staircases, where only 41% of emergency staircases were properly maintained and free of cracks, while only 35% had the appropriate strength to bear the cumulative weights of the total numbers of students. Besides, only 47% of the schools had sufficient and evenly distributed emergency exits; 76–78% of those emergency exits were free from obstacles and ready to rapidly open from the inside, leading to safe outer premises.

**Table 2:** Safety of the school's main building (Q1-Q13).

Question	Answer	Frequency	Percentage
Q1: Is the school's total area suitable for the number of students present?	Yes	9	53%
	No	8	47%
	Not present	0	0%
Q2: Is there suitable shading in the school to protect students from sunlight, rain, and dust?	Yes	14	82%
	No	2	12%
	Not present	1	6%
Q3: Are the school walls high enough to ensure that students cannot climb or jump?	Yes	15	88%
	No	1	6%
	Not present	1	6%
Q4: Are the school walls free from cracks and fractures that can cause accidents?	Yes	15	88%
	No	1	6%
	Not present	1	6%
Q5: Are the lower and upper water tanks designed and installed safely?	Yes	16	94%
	No	0	0%
	Not present	1	6%
Q6: Is there an emergency staircase extended from the upper floors leading to a safe area on the ground floor?	Yes	10	59%
	No	0	0%
	Not present	7	41%
Q7: Is the bearing strength of the emergency staircase appropriate for the total number and weight of students?	Yes	6	35%
	No	4	24%
	Not present	7	41%
Q8: Is the emergency staircase free from any affecting fractures and cracks?	Yes	7	41%
	No	3	18%
	Not present	7	41%
Q9: Are there enough emergency exits that are evenly distributed?	Yes	8	47%
	No	8	47%
	Not present	1	6%
Q10: Are the emergency exits free from obstacles and ready to open rapidly from the inside?	Yes	14	82%
	No	2	12%
	Not present	1	6%
Q11: Do the emergency exits lead to safe areas?	Yes	13	76%
	No	3	18%
	Not present	1	6%
Q12: Are the emergency exits provided with safety signs and arrows indicating their locations?	Yes	13	76%
	No	3	18%
	Not present	1	6%
Q13: Is the schoolyard free from furniture or flammable materials?	Yes	16	94%
	No	1	6%
	Not present	0	0%

Table 3 presents the data analysis of the classroom safety item, which contains 7 questions (Q14-Q20). Most of the schools (94%) have safe and even instillation of electrical wiring inside classrooms. Furthermore, 71% of the ventilation systems inside classrooms were good and sufficient. More than half (59%) of the schools have the appropriate distribution of students’ seats and sufficient walking space. In addition, 76% of schools have adequate illumination. Fortunately, there are measures to ensure that power is turned off at the end of the school day in 100% of the schools, and most of the schools (88%) have window security measures, especially on the upper floors.

**Table 3:** Classroom safety (Q14-Q20).

Question	Answer	Frequency	Percentage
Q14: Is the electrical wiring inside classrooms installed evenly and safely?	Yes	16	94%
	No	1	6%
	Not present	0	0%
Q15: Is the ventilation good and sufficient?	Yes	12	71%
	No	5	29%
	Not present	0	0%
Q16: Are the students' seats distributed appropriately with sufficient space for movement?	Yes	10	59%
	No	7	41%
	Not present	0	0%
Q17: Are there signs indicating safety instructions?	Yes	10	59%
	No	7	41%
	Not present	0	0%
Q18: Is there adequate illumination?	Yes	13	76%
	No	4	24%
	Not present	0	0%
Q19: Is there insurance that power sources are turned off at the end of the school day?	Yes	17	100%
	No	0	0%
	Not present	0	0%
Q20: Are there security measures for windows, especially on upper floors?	Yes	15	88%
	No	2	12%
	Not present	0	0%

Table 4 represents the data analysis of the laboratory safety item, which contains 13 questions (Q21-Q33). Laboratories are located on the ground floor in 65% of the studied schools. Unfortunately, 53% of the labs are not equipped with safety shelves for storing chemicals, and 41% of the labs have name labels, and safe handling instructions of hazardous materials on chemical packages or bottles. Besides, 76% of labs were not equipped with suitable safety cabinets or effective exhaust fans, and only 53% had appropriate first aid kits. On the other hand, 71% of school labs were equipped with recently checked fire extinguishers and smoke detectors.

**Table 4:** Laboratory safety (Q21-Q33).

Question	Answer	Frequency	Percentage
Q21: Is the school laboratory located on the lower floor?	Yes	11	65%
	No	1	6%
	Not present	5	29%
Q22: Are there safety shelves for storing chemicals?	Yes	8	47%
	No	0	0%
	Not present	9	53%
Q23: Is the method of storing chemicals safe (out of reach - far from sunlight and heat sources - properly packaged)?	Yes	6	35%
	No	1	6%
	Not present	10	59%
Q24: Are there suitable safety cabinets and effective exhaust fans?	Yes	3	18%
	No	1	6%
	Not present	13	76%
Q25: Is there a recently checked fire extinguisher?	Yes	12	71%
	No	0	0%
	Not present	5	29%
Q26: Is there an appropriate first aid kit?	Yes	9	53%
	No	2	12%
	Not present	6	35%
Q27: Is there an operational smoke detector?	Yes	12	71%
	No	0	0%
	Not present	5	29%
Q28: Is there an unobstructed emergency exit?	Yes	8	47%
	No	0	0%
	Not present	9	53%
Q29: Is there appropriate personal protective equipment (such as rubber gloves)?	Yes	6	35%
	No	0	0%
	Not present	11	65%
Q30: Is the electrical wiring inside the lab installed evenly and safely?	Yes	12	71%
	No	0	0%
	Not present	5	29%
Q31: Are there safety handling instructions for using chemicals?	Yes	7	41%
	No	1	6%
	Not present	9	53%
Q32: Are there labels on each chemical packaging or bottle indicating its name and its hazardous status?	Yes	7	41%
	No	0	0%
	Not present	10	59%
Q33: Is there a daily follow-up for closing the gas extensions and keys?	Yes	1	6%
	No	0	0%
	Not present	16	94%

Table 5 presents the data analysis of the school canteen safety item, which contains 7 questions (Q34-Q40). All the studied schools had safe electrical wiring in the canteens, and more than half (59%) had wooden insulators under the freezers. The fire extinguishers in the canteens were recently checked and operational in almost all schools (94%). Only 65% of the canteens had good quality ventilation systems.

**Table 5:** School canteen safety (Q34-Q40).

Question	Answer	Frequency	Percentage
Q34: Is the school canteen located on the ground floor?	Yes	15	88%
	No	2	12%
	Not present	0	0%
Q35: Is the electrical wiring inside the canteen installed evenly and safely?	Yes	17	100%
	No	0	0%
	Not present	0	0%
Q36: Is there wooden insulation under the freezer?	Yes	7	41%
	No	10	59%
	Not present	0	0
Q37: Are there separate electrical connections for the thermal appliances?	Yes	16	94%
	No	0	0%
	Not present	1	6%
Q38: Is there a recently checked and operational fire extinguisher?	Yes	16	94%
	No	1	6%
	Not present	0	0%
Q39: Is the electric oven safe and free of spilled oils and food?	Yes	1	6%
	No	0	0%
	Not present	16	94%
Q40: Is the quality of ventilation good?	Yes	11	65%
	No	6	35%
	Not present	0	0%

Table 6 represents the data analysis of the school's safety committee engagement, which contains 11 questions (Q41-Q51). All the studied schools had safety committees that conducted regular training programs for students about safety practices. In addition, proper follow-up and supervision were carried out during students' entry and exit to ensure the application of accident prevention protocols. Moreover, all schools (100%) carry out biannual check-ups and maintenance for all fire extinguishers. On the other hand, 94% of schools did not have a safe method for storing flammable materials, while 88% of the schools conducted periodic inspections after laboratory sessions to ensure the disconnection of thermal devices.

**Table 6:** Schools’ safety committees (Q41-Q51).

Question	Answer	Frequency	Percentage
Q41: Is there a school safety committee?	Yes	17	100%
	No	0	0%
	Not present	0	0%
Q42: Is there a regular training program for students about safety practices?	Yes	17	100%
	No	0	0%
	Not present	0	0%
Q43: Is the safety committee conducting educational programs for school employees (management - teachers - workers) about safety practices?	Yes	17	100%
	No	0	0%
	Not present	0	0%
Q44: Is there follow-up and observation of students' behavior during the school day?	Yes	17	100%
	No	0	0%
	Not present	0	0%
Q45: Is there an observation of students during entry and exit to prevent accidents?	Yes	17	100%
	No	0	0%
	Not present	0	0%
Q46: Is there periodic inspection after laboratory sessions to ensure the disconnection of thermal devices after use?	Yes	15	88%
	No	1	6%
	Not present	1	6%
Q47: Is there an efficient method for safely storing excess flammable materials?	Yes	1	6%
	No	0	0%
	Not present	16	94%
Q48: Are there biannual check-ups and maintenance for all fire extinguishers in the school?	Yes	17	100%
	No	0	0%
	Not present	0	0%
Q49: Is there immediate and quick contact with the school manager pertinent to existing or expected dangers?	Yes	17	100%
	No	0	0%
	Not present	0	0%
Q50: Is there a first aid kit available?	Yes	17	100%
	No	0	0%
	Not present	0	0%
Q51: Is there communication with students' parents to develop their safety awareness?	Yes	16	94%
	No	1	6%
	Not present	0	0%

Table 7 presents the data analysis of the last item of the checklist (school evacuation plan), which contains 7 questions (Q52-Q58). All the studied schools have a school evacuation plan that is practiced at least once a year. The Civil Defense phone number is placed in a visible and accessible area in 71% of the schools. Almost 82% of the schools have a safe assembly point in case of evacuation and maps or signs for guiding students during the event of an emergency evacuation. On the other hand, 94% of the schools did not have a standard operating system for disaster management.

**Table 7:** School evacuation plan (Q52-Q58).

Question	Answer	Frequency	Percentage
Q52: Is there a school evacuation plan?	Yes	17	100%
	No	0	0%
	Not present	0	0%
Q53: Is there periodic practice of the evacuation plan (at least once a year)?	Yes	17	100%
	No	0	0%
	Not present	0	0%
Q54: Is there a safe assembly area in case of evacuation?	Yes	14	82%
	No	3	18%
	Not present	0	0%
Q55: Is there a map or signs for guiding students during the event of an emergency evacuation?	Yes	14	82%
	No	3	18%
	Not present	0	0%
Q56: Is there an appropriate warning system in case of evacuation (such as a ringing bell or siren)?	Yes	16	94%
	No	1	6%
	Not present	0	0%
Q57: Is the Civil Defense phone number placed in a visible and reachable place?	Yes	12	71%
	No	4	23%
	Not present	1	6%
Q58: Is there a standard operating system for disaster management?	Yes	1	6%
	No	16	94%
	Not present	0	0%

**Fig. 1:**
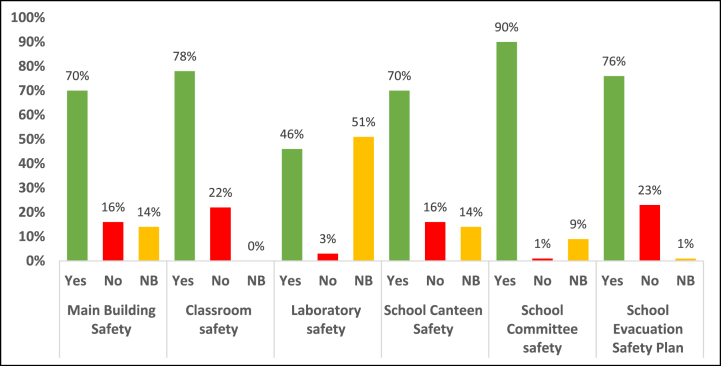
The mean percentage for each answer option of the safety preparedness items.

The mean percentage for each response for the six safety preparedness items is presented in Fig. 1.

### 3.1 Government-constructed and rented school buildings

As mentioned earlier, there are two types of government school buildings: constructed and rented. The constructed buildings are designed and built by the concerned governmental authorities, while the rented buildings are rented out by the government from private parties and are adjusted to serve as school buildings. Table 8 demonstrates the levels of safety items and the overall safety preparedness in the two types of buildings. The safety preparedness in the government-constructed is much higher than in the rentals. For example, the classroom safety in the constructed buildings is 84.7%, while in the rentals it is 47.6%. The overall safety preparedness is 74.9% and 49.4% in the constructed and rentals respectively..

**Table 8:** Safety preparedness in government-constructed and rental school buildings.

Safety Preparedness Item		Constructed	Rental
Safety of the school's main building	Yes	74.7%	53.9%
	No	9.4%	46.1%
	Not present	15.9%	0%
Classroom safety	Yes	84.7%	47.6%
	No	15.3%	52.4%
	Not present	0%	0%
Laboratory safety	Yes	56.0%	0%
	No	6.6%	0%
	Not present	37.4%	100%
School canteen safety	Yes	71.4%	61.9%
	No	14.3%	23.8%
	Not present	14.3%	14.3%
Schools' safety committees	Yes	90.3%	87.9%
	No	0.7%	3.0%
	Not present	9.0%	9.1%
School evacuation plan	Yes	79.6%	61.9%
	No	19.4%	38.1%
	Not present	1.0%	0%
	Yes	74.9%	49.4%
Overall safety preparedness	No	9.6%	24.7%
	Not present	15.5%	25.9%

## 4 Discussion

As previously mentioned, the safety checklist included six main items, where each item was composed of several questions and each question had three possible responses: Yes, No, and Not present (NP). Concerning the first item (Safety of the school's main building), 70% of all safety items are appropriately applied, while 16% are not adequately applied. One of the main shortfalls in safety preparedness in the school building, which was seen in 47% of the selected schools, is the disproportionality of the available space area to the number of enrolled students. This may increase the probability of accidents and injuries, especially during an emergency. Besides, 41% of the schools do not have emergency staircases, which means students and staff will have to use the same stairs during emergencies, and the bearing strength of the emergency stairs was found to be inappropriate for the number and total expected weight of students at 65% of the schools. Furthermore, the emergency stairs in 18% of schools are not free from fractures and cracks, which may cause severe accidents or collapse during an evacuation, especially in schools with multiple floors.

Similarly, 78% of the (Safety of the classrooms) items are adequately applied. The main shortages in this item included the absence of signs that indicate safety instructions and sufficient safe movement space in 41% of the studied schools. In addition, 24% of schools do not have adequate illumination in classrooms. Results of the third item (Lab safety) revealed that only 41% of the recommended safety items are present and appropriately applied. The main lacking safety items included the availability of safety shelves for storing chemicals, appropriate methods for keeping chemicals out of reach and far from sunlight and heat sources, suitable safety cabinets and effective exhaust fans, appropriate personal protective equipment (such as rubber gloves), labels on chemical packaging or bottles indicating the name and hazardous status, and the availability of first aid kits.

As for the school canteen safety items, 70% of the items are adequately applied in the studied schools, whereas 16% are poorly applied. The main deficiencies in this item included the absence of wooden insulators under freezers (59%) and poor ventilation. Fortunately, 90% of the school safety committee items are present in the studied schools. Most items were 100% satisfied, and the only item that was completely absent was the method for safe storage of excess flammable materials. For the last item (School evacuation plan), 76% of the items are adequately present. The main shortages in this item included the absence of a standard operating system for disaster management, the lack of visibility and accessibility to the Civil Defense phone number, and the absence of maps and signs guiding students during emergency evacuation events.

Generally, some of the studied schools are not compliant with many of the recommendations for safety and emergency preparedness due to many reasons, such as insufficient training programs for both, school staff and students, and the inappropriate characteristics of some school buildings. Some of the school buildings are rented properties, not originally designed to serve as schools, therefore, lacking in certain safety criteria. Even in some government-constructed schools, particularly the older ones, the school facilities have not been well-designed to accommodate safety preparedness requirements.

Limited studies have been conducted to evaluate safety preparedness in schools, in the Middle East in general, and in KSA in particular. For example, in KSA, Alsubaie [8] stated that many accidental tragedies have occurred over the past few years in several schools in different governorates. For instance, in Jeddah City, a school fire tragedy occurred in 2011, where two female teachers suffered deadly injuries, and over 60 students and personnel were harmed. Another fire incident occurred in a school in Jazan City in 2014 and resulted in the hospitalization of 3 students due to suffocation. The study revealed that there is a lack of healthcare professionals capable of responding to school medical emergencies and a lack of accurate data reflecting the incidence and prevalence of such accidents. Another study was conducted to measure the preparedness of schools in the city of Makkah to deal with possible earthquakes. The results of the study indicated that both, public and private schools, were not well-prepared to manage disasters such as earthquakes. As a result, one of the main recommendations of this study was to establish a department for disaster and crisis management in the MoE. This department should be responsible for preparing and delivering disaster awareness programs [16].

Worldwide, many studies have been conducted to evaluate safety preparedness in schools. For example, a recent study conducted in Vanuatu in the South Pacific Ocean concluded that school management must develop a maintenance plan, including responsible parties and budget, and inspect school facilities at the beginning of each academic year to make necessary repairs and report back to the subnational education office [17]. Another recent study was conducted in schools in Khyber Pakhtunkhwa Province, Pakistan, revealing that the efforts of preparedness in terms of emergency planning were low (40%), and 74% of schools in Pakistan failed to create school evacuation maps and routes, which are an essential part of an emergency plan as they help students and staff safely evacuate the building during an emergency [18]. In Democratic Republic (Lao PDR), an Asian tropical country, a study was conducted to inform better implementation strategies on disaster risk reduction and management in a school setting focused on fire disasters. One of the important recommendations posited in this study was the implementation of disaster risk reduction programs in schools and the impact such programs would have on improving students’ knowledge and practices [19]. In Sri Lanka, a study was conducted on several public schools to assess canteens in terms of location, safety, as well as other aspects. It was found that 88% of canteens were located in suitable places, and 66.7% had adequate ventilation [20]. Another study conducted in the Northern Region of Ghana revealed that there was a lack of teacher training and professional development, as well as inadequate teaching and learning materials when it comes to safety. The study concluded that new and consolidated efforts were needed from all stakeholders to train teachers and provide appropriate learning materials to improve disaster risk management [21].

Turan et al., conducted a study to assess the health and safety levels at secondary schools in Turkey, from the perspective of teachers and students, principals, and parents, to identify pertinent problems and constraints. The study revealed that risks to school health and safety included physical elements, such as the conditions of stairs and sinks. Additional risks included external elements such as pedestrian crossing points and traffic lights around schools [22]. Another exploratory study was conducted to assess knowledge and self-expressed practices regarding disaster management among 540 secondary school teachers in Pune City, India. The study concluded that teachers’ knowledge and self-expressed practices were not at satisfactory levels [23].

Official education bodies around the world are paying more attention to safety preparedness programs in schools. Likewise, the Saudi MoE plays a key role in planning the evaluation and training of school safety preparedness programs. However, to increase awareness and maintain safety inside schools, collaborative efforts between the MoE, students, teachers, school administrators, and school staff are necessary. The importance of establishing a formal collaborative initiative is exemplified in the cases of other countries, such as Pakistan, where school safety and preparedness are still considered a choice, rather than a mandatory operating requirement for all schools [18]. In the case of Saudi schools, safety and preparedness regulations are considered mandatory as per the directives of the MoE.

In the Dominican Republic, for example, the MoE developed a handbook series for teachers to incorporate disaster risk management in classrooms, specifically considering major hazards [17]. Nepal's Department of Education has guidelines for designing schools to fit a range of social, physical, and environmental contexts. It incorporates a multi-hazard approach for safe site selection, design, construction, and maintenance of facilities [24]. The MoE in Bangladesh conducts evacuation and first aid drills biannually, whereas the Philippines' Department of Education conducts such drills every quarter [17]. In Costa Rica, the MoE has trained 120 cadres since 2003, to ensure they can impart knowledge about disasters across various regions. As a result, these cadres have managed to coach 6000 educators [25]. The crisis management plan for St. Agnes Catholic School in Illinois, USA, outlined that the principal would organize educator training for cardiopulmonary resuscitation (CPR), first aid, and the use of fire extinguishers [26]. Fiji's educational authority and National Disaster Management office developed many standard operating procedures for emergencies and safety procedures for schools through national broadcasts on public television channels to ensure that students, staff, and teachers watch and practice emergency response protocols [17]. A school-based full-scale emergency exercise led by the Singaporean Department of Civil Defense is conducted regularly involving school administrators, teachers, students, as well as parents. Scale fire and evacuation drills were likewise conducted to supplement the full-scale exercise [27]. In the UK, a study was done to examine classrooms that are lit with high fluorescent lighting in a sample of schools. Results of this study showed that the mean illuminance (from excessive daylight and artificial lighting) exceeded the recommended design illumination in 88% of classrooms, and in 84% of them, it exceeded levels beyond which visual comfort decreases. Such illumination defects may cause headaches and impaired visual performance [28].

The safety preparedness in the government-constructed schools is much higher than in the rentals. This may be related to the clear difference in the design of the two building types. The overall school size, number of classrooms, roads inside the building, facilities (such as toilets, labs, canteen) and the playground in the governmental-constructed buildings are usually more in number and wider in size than the rental type. In addition, most of the governmental constructed buildings are will-designed and constructed under the supervision of expert engineers, other than the rented schools which were houses but converted into schools to accommodate the increasing number of students.

## 5 Conclusion

The safety preparedness in the studied schools ranged between 70 and 90%. Some of the safety practices were adequately applied, others were poorly applied, and certain safety items were completely absent. Generally, some of the studied schools were not compliant with many of the recommendations for safety and emergency preparedness due to many reasons, such as insufficient training programs for school staff and students and the lack of safety characteristics at some school buildings. Some school buildings are rented houses that do not meet the safety criteria required for schools. Even in some public schools constructed by the government, particularly the older ones, school facilities have not been well-designed to accommodate the safety requirements. School safety committees should assist in the preparation of school safety management plans and collaborate with other government bodies to effectively improve school safety conditions.

## Strengths and limitations

In Saudi Arabia, male and female upper-level schools are segregated. Although several studies have been conducted to evaluate the safety preparedness of male schools, there has been a shortage in similar studies addressing female schools. This study is the first to address such an issue and serves as a reference point for future safety preparedness studies, particularly in Saudi female schools, and Middle Eastern schools in general. In addition, this study will help in raising awareness among students and school personnel regarding safety criteria and practices. The study sample is not exhaustive, due to the limited accessibility of male researchers to female schools.

## Author contribution statement

Naof F. Al-Ansary, Mahmoud F. El-Sharkawy, Sana A. Alsulaiman: Conceived and designed the experiments; Performed the experiments; Analyzed and interpreted the data; Contributed reagents, materials, analysis tools or data; Wrote the paper

## Data availability statement

Data will be made available on request.

## Additional information

No additional information is available for this paper.

## Declaration of competing interest

The authors declare the following financial interests/personal relationships which may be considered as potential competing interests: Mahmoud Fathy Mohamed Elsharkawy reports administrative support was provided by 10.13039/501100015090Imam Abdulrahman Bin Faisal University. Mahmoud Fathy Mohamed Elsharkawy reports a relationship with Imam Abdulrahman Bin Faisal University that includes: employment. Mahmoud Fathy Mohamed Elsharkawy has patent pending to Licensee. No conflict of Interest.

## References

[1] Schroeder K., Kubik M.Y. (2019). Policy, systems, and environmental approaches to a healthy school environment. J. Adolesc. Health.

[2] Mege C.A. (2014). http://erepository.uonbi.ac.ke/handle/11295/77873.

[3] Lawrence A.S.A., Vimala A. (2012). School environment and academic achievement of standard IX students. J Edu Ins Stu World.

[4] Borse N.N., Gilchrist J., Dellinger A.M., Rudd R.A., Ballesteros M.F., Sleet D.A. (2008). CDC childhood injury report: patterns of unintentional injuries among 0- to 19-year-olds in the United States, 2000–2006. Fam. Community Health.

[5] Mooney C.N., Ross J.G., Moloney M. (2010). Sustaining a safe and healthy school environment every day. Momentum.

[6] Minnesota Department of Public Safety Division of Homeland Security and Emergency Management (HSEM) (2005).

[7] Who (2017). https://apps.who.int/iris/handle/10665/42683.

[8] Alsubaie A. (2017). School safety and emergency preparedness in Saudi Arabia: a call for effective action. Int J Res MedSci.

[9] Bendak S. (2006). Evaluation of school safety in Riyadh. Int. J. Inj. Control Saf. Promot..

[10] Vicario A.D. (2017). Practices that promote comprehensive school safety. New Trends and Issues Proceedings on Humanities and Social Sciences.

[11] New Jersey Department of Education (2011). School safety and security plan review checklist. https://www.state.nj.us/education/schools/security/req/checklist.pdf.

[12] Vermont Department of Education (2005).

[13] Ministry of Home Affairs (2004). School safety. National disaster management division, Ministry of Home Affairs India. https://nidm.gov.in/PDF/safety/school/link1.pdf.

[14] Delaware Department of Education (2002). School safety audit guidelines. http://www.doe.state.de.us/docs/pdf/dedoe_schoolsafeaudit.pdf.

[15] Ministry of Education (2018). https://departments.moe.gov.sa/SchoolSafety/FileLibrary/Documents/DaleelAHSA.pdf.

[16] Momani N.M., Salmi A. (2012). Preparedness of schools in the Province of Jeddah to deal with earthquake risks. Disaster Prev. Manag..

[17] Paci-Green R., Varchetta A., McFarlane K., Iyer P., Goyeneche M. (2020). Comprehensive school safety policy: a global baseline survey. Int. J. Disaster Risk Reduc..

[18] Shah A.A., Ye J., Pan L., Ullah R., Ali Shah S.I., Fahad S., Naz S. (2018). Schools' flood emergency preparedness in khyber Pakhtunkhwa Province, Pakistan. IntJ Disaster Risk Scie.

[19] Kanyasan K., Nonaka D., Chatouphonexay A., Hernandez P.M., Kounnavong S., Kobayashi J. (2018). Implementation of disaster risk reduction and management policies in a school setting in Lao PDR: a case study. Trop. Med. Health.

[20] Weerasinghe M., Bandara S., Sanoon M. (2017). Service quality of school canteens: a case study from the Western Province, Sri Lanka. Ceylon J. Med. Sci..

[21] Apronti P., Saito O., Otsuki K., Kranjac-Berisavljevic G. (2015). Education for disaster risk reduction (DRR): linking theory with practice in Ghana's basic schools. Sustainability.

[22] Turhan M., Turan M. (2012). Safety in secondary education institutions educational administration. Theor. Pract..

[23] Joshi S.G. (2014). Knowledge and practices of school teacher regarding disaster management. IntJ Health Sys DisManag.

[24] Varchetta A. (2019). Evaluating comprehensive school safety through a global baseline survey of disaster risk reduction policies in the education sector. https://cedar.wwu.edu/cgi/viewcontent.cgi?article=1903&context=wwuet.

[25] Wisner B. (2006). https://www.researchgate.net/publication/44836374.

[26] Agnes St (2011). St. Agnes Catholic school crisis management plan. https://docplayer.net/8798385-St-agnes-catholic-school-crisis-management-plan.html.

[27] Reyes M.L., Diopenes V.E., Co R., Berse P. (2011). http://www.preventionweb.net/files.

[28] Winterbottom M., Wilkins A. (2009). Lighting and discomfort in the classroom. J. Environ. Psychol..

